# Evaluation of a quintuple-gene-deleted PRV vector expressing PEDV S1: safety and immunogenicity in rabbits, mice, and piglets

**DOI:** 10.3389/fmicb.2026.1878909

**Published:** 2026-07-08

**Authors:** Junda Li, Jiadeng Jiang, Ruhai Guo, Xiaofan Wang, Litian Shi, Saba Nasir, Yining Zhang, Wenli Shi, Yanqing Jia, Xinglong Wang

**Affiliations:** 1 College of Veterinary Medicine, Northwest A&F University, Yangling, Shaanxi, China; 2 Department of Animal Engineering/Shaanxi Engineering Research Center of Animal Disease Prevention and Control/The Youth Innovation Team of Shaanxi Universities, Shaanxi A&F Technology University, Yangling, Shaanxi, China

**Keywords:** CRISPR/Cas9 gene editing, immunogenicity, porcine epidemic diarrhea virus, pseudorabies virus, vaccine

## Abstract

Pseudorabies virus (PRV) and Porcine epidemic diarrhea virus (PEDV) are currently co-circulation among swine herd with multiple variant strains, which cause severe economic losses to the global swine industry. In the present study, a recombinant PRV-based vaccine candidate was constructed backbone of a PRV variant via deletion of five genes (gI, gE, US2, US9, and TK) and insertion of the S1 gene derived from a PEDV G2b strain, and the resulting recombinant virus was generated as rPRV-Δ5-S1. The safety of the quintuple-gene-deleted PRV vector (rPRV-Δ5) was firstly evaluated in rabbits and compared with the previously generated triple-gene-deleted vector (rPRV-Δ3). Rabbits inoculated with rPRV-Δ5 exhibited milder clinical manifestations, lower viral loads in tissues, and fewer histopathological lesions, indicating an improved safety profile. Subsequent immunization trials in mice demonstrated that rPRV-Δ5-S1 induced PEDV S1-specific antibodies and PEDV-neutralizing antibodies, which were higher than those observed in the commercial inactivated PEDV vaccine group. Furthermore, rPRV-Δ5-S1 elicited higher neutralizing antibody titers against PRV variants than the Bartha-K61 vaccine and provided complete protection against lethal challenge with the PRV variant. To further validate the immunogenicity and protective efficacy of rPRV-Δ5-S1, challenge experiments were conducted in piglets. The results showed that piglets immunized with rPRV-Δ5-S1 developed detectable antibody responses against both PRV and PEDV. After challenge with the PEDV G2b variant, rPRV-Δ5-S1-immunized piglets exhibited reduced diarrhea severity, decreased viral shedding, alleviated intestinal lesions, and improved weight gain compared with control animals. Collectively, these findings demonstrate that the quintuple-gene-deleted PRV vector exhibits an improved safety profile and can serve as an effective platform for heterologous antigen delivery. The recombinant virus rPRV-Δ5-S1 can induced immune responses against both PRV and PEDV in multiple animal models, which supports its potential as a bivalent vaccine candidate against these two economically critical swine pathogens.

## 1 Introduction

PEDV has caused substantial economic losses to the global swine industry, particularly following the emergence and rapid spread of highly virulent variant strains in China since 2010 (Lei et al., 2024). Two main genotypes, G1 and G2, are currently prevalent in China, with G2 strains becoming predominant after 2015 (Liang et al., 2020). The spike (S) protein of PEDV serves as the primary antigenic target for vaccine development due to its critical role in viral entry and in inducing neutralizing antibodies (Luo et al., 2024). However, existing commercial vaccines, including inactivated and attenuated live formulations, face limitations such as insufficient cross-protection, virulence reversion risks, and suboptimal mucosal immunity (Wei et al., 2024).

Since 2011, emerging virulent PRV strains have caused severe pseudorabies (PR) outbreaks in vaccinated swine populations across China. Although classical vaccines, such as Bartha-K61, have played an important role in controlling pseudorabies, their protective efficacy against currently circulating PRV variants is limited, highlighting the urgent need for the development of next-generation vaccines based on contemporary field strains (Tan et al., 2021). Concurrently, the exceptionally large genome of PRV exhibits high plasticity, allowing for the insertion of foreign genes without significantly compromising viral infectivity or intrinsic immunogenicity (Kimman et al., 1992). To address these challenges, we developed a recombinant quintuple-gene-deleted PRV vector derived from a currently circulating PRV variant and engineered it to express the PEDV S1 antigen. This strategy aims to improve antigenic matching against contemporary PRV strains while simultaneously inducing immune responses against PEDV.

The CRISPR/Cas9 system is a powerful tool for efficiently constructing attenuated PRV vaccines, enabling targeted deletion of virulence genes (such as gE, gI, TK) and insertion of antigen-encoding sequences (Luo et al., 2022; Lv et al., 2021). The PRV genome is a double-stranded DNA molecule of approximately 145 kb, which is divided into the unique long (UL) and the unique short (US) regions, encoding proteins essential for viral structure and host infection (Díaz et al., 2021). The safety of PRV as a vaccine vector can be enhanced by knocking out specific virulence genes. For example, deletion of the TK gene (UL23) has been shown to reduce neurovirulence and latency while enhancing host immune responses (Wang et al., 2021; Hsu et al., 2024; Deng et al., 2022).

Deletion of gE/gI (US7/US8) disrupts neuronal spread through US9-mediated axonal transport, resulting in attenuated vaccine candidates that have demonstrated protective efficacy in pigs (Lv et al., 2021; Diwaker et al., 2020; Scherer et al., 2020; Li et al., 2021). Additionally, deletion of US9 has been associated with reduced virulence and impaired spread within the nervous system (Tan et al., 2021; Lin et al., 2020). Previous studies have suggested that US2 functions as an antagonist of STING-dependent interferon signaling, and its deletion may contribute to attenuation and enhanced innate immune recognition (Kong et al., 2024; Delva et al., 2022). Furthermore, the large PRV genome provides sufficient capacity for the insertion of heterologous antigens (e.g., CSFV E2), making it an attractive platform for multivalent vaccine development (Lu et al., 2024; Wang et al., 2024). Recombinant PRV vectors have been reported to induce both humoral and cellular immune responses, including neutralizing antibody production and T-cell activation (Lu et al., 2024).

In this study, a live-attenuated recombinant PRV vaccine candidate, named rPRV-Δ5-S1, was generated by deleting five virulence-associated genes (gE, gI, TK, US9, and US2) and inserting the S1 gene derived from a PEDV G2b strain. Given the high susceptibility of rabbits to PRV infection (Sehl and Teifke, 2020; Cao et al., 2022), rabbits were used to evaluate the safety of the recombinant vector. The immunogenicity and PRV-related protective efficacy of rPRV-Δ5-S1 were subsequently assessed in a mouse model, while piglets were used to obtain preliminary evidence of PEDV-related protection in the natural host. The overall objective of this study was to evaluate the safety, immunogenicity, and potential of rPRV-Δ5-S1 as a bivalent PRV/PEDV vaccine candidate.

## 2 Materials and methods

### 2.1 Cells, viruses, bacterial strains, and plasmids

BHK-21 (ATCC CCL-10™), Vero C1008 (E6) (ATCC CRL-1586™), and PK-15 (ATCC CCL-33™) cells were obtained from the American Type Culture Collection (ATCC) and maintained in Dulbecco’s Modified Eagle’s Medium (DMEM; Gibco) supplemented with 10% heat-inactivated fetal bovine serum (FBS; Sigma-Aldrich) and 1% penicillin–streptomycin solution (Thermo Fisher Scientific, Waltham, MA, USA). Cells were cultured at 37 °C in a humidified incubator containing 5% CO₂.

The following viruses were maintained at −80 °C in the College of Veterinary Medicine, Northwest A&F University: (1) PEDV-XN-2022, a genotype G2b field isolate maintained in our laboratory; (2) PRV variant strain PRV-SX10-2015 (GenBank accession no. PP097194.1); (3) recombinant PRV strain rPRV-ΔTK-eGFP-ΔgE/gI-mCherry (hereafter referred to as rPRV-Δ3), a previously described TK/gE/gI-deleted recombinant derived from PRV-SX10-2015 (Luo et al., 2022); (4) the commercial PRV vaccine strain Bartha-K61 (Weimeirui®, Harbin Pharmaceutical Group Bio-vaccine Co., Ltd., China); and (5) a commercial inactivated PEDV vaccine (Tianfuqing®, Tiankang Biological Pharmaceutical Co., Ltd., China).

Chemically competent *Escherichia coli* DH5α and BL21(DE3) cells were purchased from Thermo Fisher Scientific.

The CRISPR/Cas9 nickase plasmid vector pX335-U6-Chimeric_BB-CBh-hSpCas9n(D10A) (Addgene plasmid #42335) was used as the backbone for construction of pX335-dualU6-TagBFP, which contains an additional U6-driven sgRNA expression cassette and a TagBFP reporter (Luo et al., 2022). Previously generated plasmids included pUC19-TK-CMV-EGFP-polyA and the CRISPR/Cas9 vectors pX335-TK-sgRNA-32/955 and pX335-gI-sgRNA-18/38 (Luo et al., 2022). The pET-30a expression vector and pMD™19-T cloning vector were purchased from Sigma-Aldrich and Takara Biotechnology, respectively. All plasmids were stored at −80 °C until use.

### 2.2 Antigen detection method based on PEDV M gene

A SYBR Green I-based reverse transcription quantitative polymerase chain reaction (RT-qPCR) assay targeting the PEDV M gene was established and used for PEDV viral load quantification. Primer sequences are listed in Table 1. Standard plasmid construction, assay optimization, specificity testing, sensitivity evaluation, and standard curve generation are described in Supplementary Figure S1. The assay was subsequently used for quantification of PEDV genomic RNA in rectal swab samples.

**Table 1:** Primers used in this study.

Target gene	Primer sequence (5′-3′)
PEDV-qPCR-F	AGAGGGCTATAAGGTTGCTA
PEDV-qPCR-R	GTCCGTAGACGATTGTTGTA
CMV-S1-F	AGGTCTATATAAGCAGAGCTGCTAGCGCCACCATGAAGTCTTTAACTTACTTCTGGTTG
S1-T2A-eGFP-R	CTGCCCTCTCCACTGCCACGCGTCTAAAGTTGGTGGGAATACTAATATTCCCA
gI LA-F	ACCATGATTACGCCAAGCTTCGCGATTCCCCCCTCTCTCTCA
gI LA-R	ACTAGTCAATAATCAATGCCCTACGGACCGGGCTGCGCTTTTA
CMV-F	GGCATTGATTATTGACTAGTTAT
CMV-R	AGCTCTGCTTATATAGACCT
CMV-mCherry-F	ATATAAGCAGAGCTGCTAGCGCCACCATGGTGAGCAAGGGCGAG
mCherry-SV40polyA-R	AGAGATCCTGCCGTCTAGGATAAGATACATTGATGAGTTTGGACAAACC
US2- RA-F	TCCTAGACGGCAGGATCTCTC
US2- RA-R	ACGACGGCCAGTGAATTCGCTCTTTGGGGAGGAGTGC
TK-Del-273-F	CGCACTCTGTTCGACACGGA
TK-Del-273-R	GCTGATGTCCCCGACGATGA
gI/gE-Del-441-F	GGGAAGATAGCCATGGTGCT
gI/gE-Del-441-R	CGGACGGAGATAAAACGCCA
US9/US2-Del-347-F	GCTGGTCATCTGCTCGCTG
US9/US2-Del-347-R	CATCAGCGTGACCACGGTGA
Cherry-463-F	CCGACATCCCCGACTACTTG
Cherry-463-R	TTGTACAGCTCGTCCATGCC
eGFP-402-F	AGGACGACGGCAACTACAAG
eGFP-402-R	GTCCATGCCGAGAGTGATCC
gD-qF	TTATCGAGTACGCCGACTGC
gD-qP	ACCACGCCGATGTGGTG
gD-qR	CCTCCGTGGGGAACATGTAG
sgRNA-gI-18-F	CACCGCATCGACGCCGGTACTGCGG
sgRNA-gI-18-R	AAACCCGCAGTACCGGCGTCGATGC
sgRNA-gI-38-F	CACCGCGACGTGACCCGGCTCCCCG
sgRNA-gI-38-R	AAACCGGGGAGCCGGGTCACGTCGC
sgRNA-US2-612-F	CACCGGACCCGCGCGAACATGGCG
sgRNA-US2-612-R	AAACCGCCATGTTCGCGCGGGTCC
sgRNA-US2-699-F	CACCGCACTCCCAGATCGTGACCCG
sgRNA-US2-699-R	AAACCGGGTCACGATCTGGGAGTGC

### 2.3 Protein expression and polyclonal antibody preparation

To generate PEDV S1-specific polyclonal antibodies, a recombinant PEDV S1 protein fragment (amino acids 405–789) was expressed in *Escherichia coli* BL21(DE3) using the pET-30a expression system. The target region was selected based on transmembrane domain and B-cell epitope predictions using DeepTMHMM 1.0 and SVMTriP. Recombinant protein expression, purification, and refolding procedures are described in Supplementary Figure S1.

Purified recombinant S1 protein was emulsified with Freund’s adjuvant and used to immunize five female BALB/c mice by subcutaneous injection at 14-day intervals. Serum samples were collected 2 weeks after the final immunization and used as PEDV S1-specific polyclonal antibodies for subsequent Western blot analyses. Antibody characterization is described in Supplementary Figure S1.

### 2.4 Construction of sgRNAs and recombinant transfer plasmids

sgRNAs targeting the PRV US2 gene were designed using the online tool CRISPOR (CRISPOR, 2022). A dual-sgRNA strategy targeting the gI and US2 loci was employed to generate a large genomic deletion encompassing the intervening gE and US9 genes.

For PEDV S1 insertion, the S1 coding sequence derived from PEDV-XN-2022 was amplified by reverse transcription PCR (RT-PCR) and cloned into the donor plasmid pUC19-TK-CMV-eGFP-polyA, generating pUC19-TK-CMV-PEDV(S1)-T2A-eGFP-polyA. To facilitate deletion of the gI/gE/US9/US2 region, a donor plasmid containing PRV homologous arms flanking a CMV-mCherry expression cassette was constructed and designated pUC19-gI/US2-CMV-mCherry-polyA.

All recombinant plasmids were verified by Sanger sequencing. Primer sequences and sgRNA target sites are listed in Table 1.

### 2.5 Rescue and purification of recombinant virus strains

Recombinant viruses were generated using CRISPR/Cas9-mediated genome editing combined with homologous recombination. BHK-21 cells were co-transfected with sgRNA/Cas9 plasmids and the corresponding donor plasmids. Twenty-four hours later, cells were infected with PRV-SX10-2015 at a multiplicity of infection (MOI) of 0.01.

Following the appearance of cytopathic effects, recombinant viruses were isolated by plaque purification under fluorescence microscopy. PCR and sequencing were used to confirm the expected genomic modifications.

Deletion of the gI/gE/US9/US2 region generated the intermediate recombinant virus rPRV-ΔgI-US2-mCherry. Subsequently, the TK locus was replaced with either an eGFP expression cassette or a PEDV S1-T2A-eGFP cassette to generate rPRV-Δ5 and rPRV-Δ5-S1, respectively. At least three rounds of plaque purification were performed until homogeneous fluorescence phenotypes were obtained. Following plaque purification and PCR verification, two recombinant viruses were obtained: rPRV-Δ5 and rPRV-Δ5-S1.

### 2.6 Growth kinetics

The recombinant viruses rPRV-Δ5-S1, rPRV-Δ5, rPRV-Δ3, and the parental strain PRV-SX10-2015 were inoculated into PK-15 cells (90% confluency) at a multiplicity of infection (MOI) of 0.01. Culture supernatants were collected at 0, 12, 24, 36, 48, 60, and 72 h post-infection (hpi). The harvested viral supernatants were subjected to three freeze–thaw cycles and subsequently inoculated onto PK-15 cell monolayers in 96-well plates. A one-step growth curve was generated, and viral titers were determined as the 50% tissue culture infective dose (TCID50) using the Reed–Muench method. Growth kinetics experiments were performed in three independent biological replicates, and mean viral titers were used to generate the growth curves.

### 2.7 Genetic stability

The genetic stability of recombinant viruses rPRV-Δ5-S1 and rPRV-Δ5 was assessed through serial passaging in PK-15 cells for 20 generations (MOI = 0.1). PCR analyses were performed at passages F5, F10, and F20 confirmed the deletion of TK, gI/gE/US9/US2 genes. It also confirmed the stable insertion of the PEDV(S1) gene. Genomic DNA from the parental PRV-SX10-2015 strain served as the positive control. Nuclease-free water was used as the negative control.

### 2.8 Western blot analysis

The recombinant viruses rPRV-Δ5-S1, rPRV-Δ5, and parental strain PRV-SX10-2015 were used to infect PK-15 cells. After 36 h of culture in DMEM supplemented with 2% FBS, protein samples were collected. Western blot analysis was performed using PEDV S1 polyclonal antibody (prepared in section 2.3, 1:1000) and mouse anti-*β*-tubulin monoclonal antibody (Proteintech, 1:1000) as primary antibodies. HRP-conjugated goat anti-mouse IgG secondary antibody (1:5000 dilution) was applied. Following three washes with TBST, protein bands were visualized using Clarity Western ECL substrate (DiNing) and detected via a chemiluminescence imaging system.

### 2.9 Safety evaluation in a rabbit model

To evaluate and compare the safety of the recombinant PRV vectors, a sensitive rabbit model was employed. Twenty healthy, 30-day-old weaned rabbits (Chengdu Dossy Experimental Animals Co., Ltd.) were randomly assigned to four groups (*n* = 5 per group): (1) rPRV-Δ3, inoculated with the triple-gene-deleted virus; (2) rPRV-Δ5, inoculated with the quintuple-gene-deleted virus; (3) Mock, inoculated with sterile DMEM (negative control); and (4) PRV-SX10-2015, inoculated with the wild-type strain (positive control). All inoculations were administered intramuscularly at a dose of 1 × 105 TCID50 in 0.5 mL (or an equivalent volume of DMEM for the Mock group) on day 0. All groups were housed separately under standard laboratory conditions with ad libitum access to food and water. All rabbits were monitored daily for 21 days for clinical signs, with a particular focus on neurological symptoms. At the experimental endpoint (day 21), all surviving rabbits were euthanized for comprehensive sampling. During necropsy, major organs (brain, lung, liver, spleen) were examined for gross lesions. For histopathological analysis, tissue samples were fixed in 10% neutral buffered formalin, embedded in paraffin, sectioned, and stained with hematoxylin and eosin (H&E). For virological analysis, parallel tissue samples were immediately snap-frozen and stored at −80 °C for subsequent viral nucleic acid extraction and Real-time quantitative PCR (qPCR) to determine tissue viral loads.

### 2.10 Immunization and challenge trial in mice

A mouse model was employed to evaluate the safety and immunogenicity of rPRVs, with the immunization and challenge protection flow chart detailed in Figure 4A. Thirty-five 6-week-old female Kunming mice obtained from Chengdu Dossy Experimental Animals Co., Ltd. were randomly divided into seven groups (*n* = 5 per group). Female Kunming mice were used to minimize variability associated with aggressive behavior and territorial fighting commonly observed in group-housed male mice and to maintain experimental consistency across groups. Six groups received intramuscular injection in the hind limbs (0.1 mL/dose) with: (1) parental strain PRV-SX10-2015 (1 × 105 TCID50/0.1 mL); (2) recombinant strain rPRV-Δ5-S1 (same dose); (3) recombinant strain rPRV-Δ5 (same dose); (4) PRV Bartha-K61 vaccine (same dose); (5) PEDV inactivated vaccine (same dose); (6) DMEM. (7) Mock group, receiving no treatment. Booster immunization was administered 14 days after the primary immunization. All groups were housed separately under standard laboratory conditions with ad libitum access to food and water. Clinical signs and survival rates were monitored daily. On day 28 post-initial immunization, surviving mice were intramuscularly challenged with PRV-SX10-2015 (0.1 mL, 1 × 105 TCID50/mL in DMEM) and monitored for clinical signs over a 7-day observation period. The PEDV inactivated vaccine group served as a serological control, whereas the Mock group served as an uninfected control for pathological and virological analyses. After euthanasia, brain, liver, spleen, and lung tissues were collected for viral load quantification and histopathological analysis. Blood samples were obtained from the retro-orbital sinus at 0, 7, 14, 21, 28, and 35 days post immunization (dpi) without anticoagulant. Serum was separated by centrifugation (3,000 × g, 10 min, 4 °C) and stored at −80 °C.

**Figure 1:**
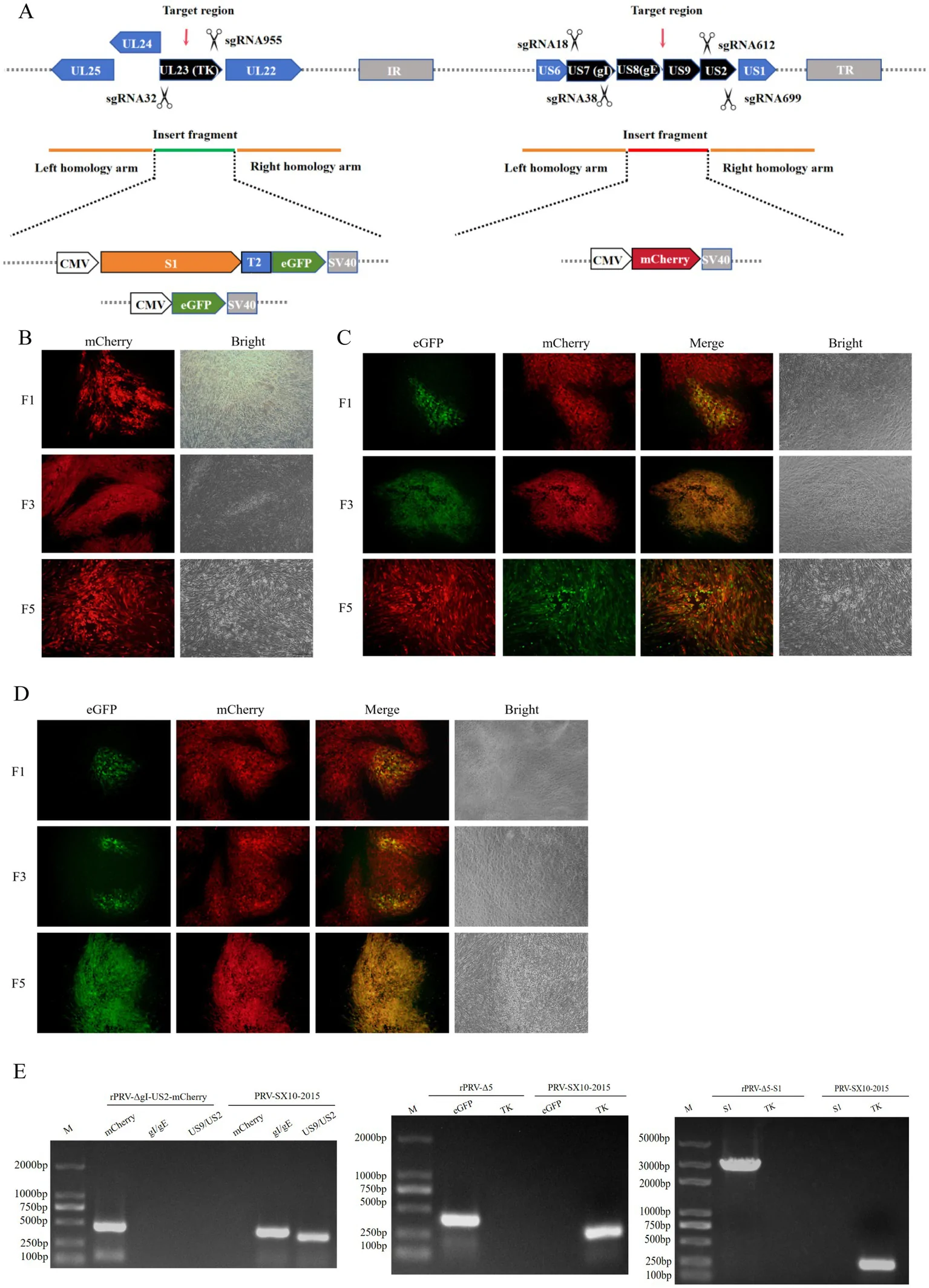
Construction of the recombinant virus rPRV-Δ5 and rPRV-Δ5-S1. **(A)** Construction flowchart. **(B)** Purification of rPRV-ΔgI-US2-mCherry observed under microscopy (100 × magnification). **(C)** Purification of rPRV-Δ5 observed under microscopy (100 × magnification). **(D)** Purification of rPRV-Δ5-S1 observed under microscopy (100 × magnification). **(E)** PCR identification of TK, gI/gE, and US9/US2 deletions, and mCherry, eGFP, and PEDV (S1) insertions in recombinant viruses.

### 2.11 Immunization and challenge trial in piglets

A piglet model was employed to obtain preliminary evidence regarding the safety, immunogenicity, and PEDV challenge-associated outcomes of rPRV-Δ5-S1. The experimental design is illustrated in Figure 6A. Nine 2-week-old PRV/PEDV double-negative healthy Yorkshire piglets were purchased from a pig farm in Yangling, Shaanxi, China, and were randomly assigned to three groups (*n* = 3 per group). The animals were housed independently in isolation units with consistent environmental conditions to minimize stress. Two groups received Houhai acupoint (GV 1) injection (2 mL/dose) of: (1) recombinant virus rPRV-Δ5-S1 (titer:1 × 106 TCID50/mL); (2) equivalent volume of DMEM medium. Vaccination was administered via the Houhai (GV1) route, which is widely used for vaccine delivery in swine production systems in China. The blank control group was not challenged and served as a baseline reference. Animals were maintained under standard conditions with ad libitum access to feed and water. Clinical signs and survival rates were monitored daily. Booster immunization was administered 21 days post-initial infection using identical protocols. On day 35 post-infection, piglets in the experimental and DMEM groups were orally challenged with 2 mL PEDV-XN-2022 (G2b) (106 TCID50/mL). Body weight, mental status, feeding behavior, and rectal temperature were recorded daily alongside rectal swabs collection. All piglets were euthanized 14 dpc (days post-challenge), and intestinal tissues were collected for pathological analysis. Serum was separated by centrifugation (3,000 × g, 10 min, 4 °C) at 0, 7, 14, 21, 28, 35, and 42 dpi and stored at −80 °C.

**Figure 2:**
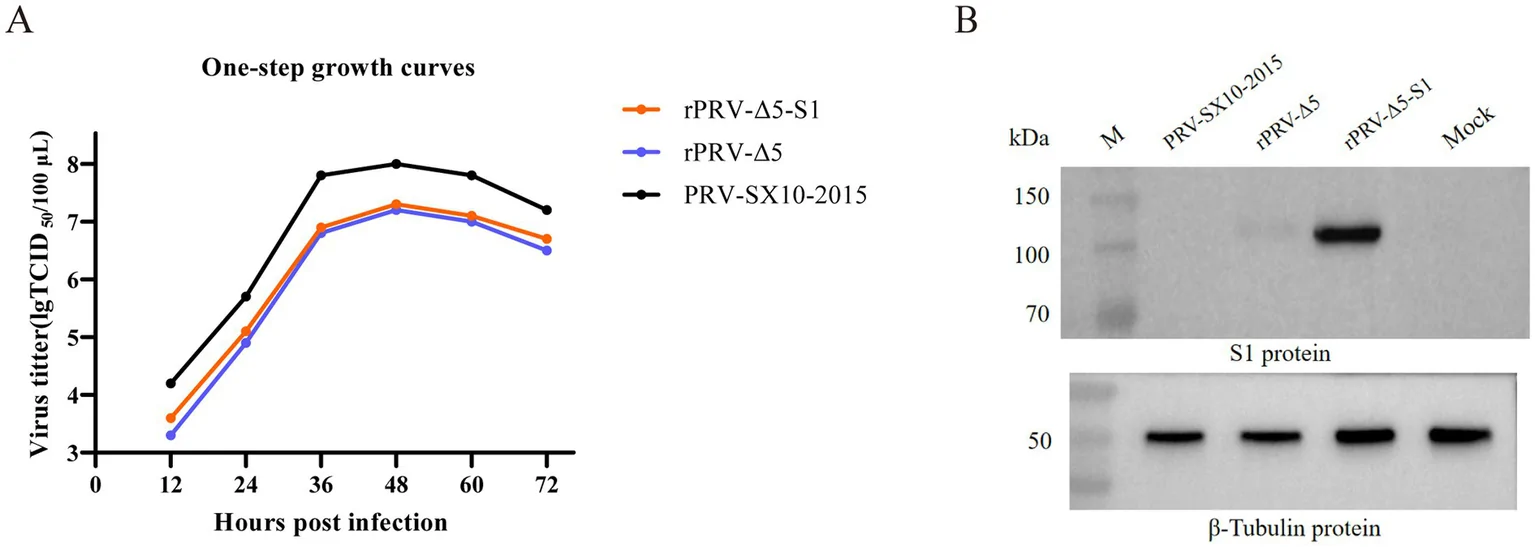
Biological characteristics of recombinant rPRV-Δ5 and rPRV-Δ5-S1. **(A)** Growth curves of the parental strain PRV-SX10-2015 and the recombinant virus strains rPRV-Δ3, rPRV-Δ5, and rPRV-Δ5-S1. Growth kinetics experiments were performed in three independent biological replicates, and mean viral titers are presented. **(B)** Western blotting of protein in BHK-21 cells infected with the recombinant viruses. Mock, lysates from uninfected BHK-21 cells.

### 2.12 Enzyme-linked immunosorbent assay (ELISA)

Humoral immune responses were evaluated by ELISA. The detection methods varied by species and antibody type, as detailed below.

For porcine samples:

PRV-gB-specific IgG antibodies: Detected using a commercial ELISA kit (Beijing Yisen Biotechnology Co., Ltd.) following the manufacturer’s instructions.
PEDV-specific IgA antibodies: Detected using a commercial ELISA kit (Shanghai Enzyme-linked Biotechnology Co., Ltd.) following the manufacturer’s instructions.
PEDV S1-specific IgG antibodies: An indirect ELISA was developed for the detection of PEDV-specific IgG antibodies (See Supplementary Figure S1). Briefly, microtiter plates were coated with purified PEDV S1 protein overnight at 4 °C, then blocked with 5% non-fat milk in PBST for 2 h at 37 °C. Serum samples were incubated for 1 h at 37 °C, followed by HRP-conjugated goat anti-pig IgG (1:5000) for 1 h at 37 °C. The reaction was developed with TMB substrate (Solarbio) for 15 min at 37 °C, terminated with 2 M H₂SO₄, and OD₄₅₀ was measured. The cut-off value (OD₄₅₀ ≥ 0.338) was defined as the mean OD₄₅₀ of 40 negative porcine sera plus three standard deviations.

For murine samples:

PRV-gB-specific IgG antibodies: Detected using a commercial ELISA kit (Beijing Yisen Biotechnology Co., Ltd.), with a modification: HRP-conjugated goat anti-mouse IgG (1:5000) was used as the secondary antibody instead of the kit-provided conjugate. All other procedures followed the manufacturer’s instructions.
PEDV S1-specific IgG antibodies: Detected using an in-house indirect ELISA, with the same protocol as for porcine samples, except that HRP-conjugated goat anti-mouse IgG (1:5000) was used as the secondary antibody. The cut-off value (OD₄₅₀ ≥ 0.198) was established using the identical method based on negative murine sera.

### 2.13 Neutralizing antibody assay

BHK-21 cells were used for PRV neutralization assays, whereas Vero cells were used for PEDV neutralization assays. BHK-21 or Vero cells were cultured in 96-well plates until achieving ≥ 90% monolayer density, followed by PBS washing and preparation for use. Serum samples were collected from vaccinated and control mice at 14 and 28 days post-vaccination, and from vaccinated and control piglets at 21 and 35 days post-vaccination. After inactivation at 56 °C for 30 min, sera underwent serial 2-fold dilutions (1:2 to 1:256) in 96-well plates with 8 replicates per dilution (final volume 50 μL). Control wells included serum toxicity controls, positive serum controls, virus-negative controls, virus titration regression controls (200–0.2 TCID50/well, prepared by serial 10-fold dilution of the challenge virus and included as back-titration controls for assay validation), and cell-only controls. PRV-SX10-2015 or PEDV-XN-2022 (G2b) strains were diluted to 200 TCID50/50 μL and mixed with diluted sera, then incubated at 37 °C for 1 h. The mixture was transferred to pretreated cell plates, adsorbed for 1 h, washed thrice with PBS, and supplemented with serum-free DMEM. Neutralizing antibody titers (NT50) were calculated using the Reed–Muench method based on cytopathic effect (CPE) observed at 72 h post-inoculation.

### 2.14 Viral load quantification

Viral load was determined in murine brain, lung, liver, and spleen tissues (0.1 g each) and piglet rectal swab samples. Viral nucleic acids were extracted using a Viral Nucleic Acid Extraction Kit (TIANGEN). PRV genome copies were quantified by qPCR targeting the gD gene as previously described (Li et al., 2025) whereas PEDV genome copies were quantified by M gene RT-qPCR using the assay established in the present study (Supplementary Figure S1). Primer sequences are listed in Table 1.

### 2.15 Diarrhea symptom scoring

Diarrhea symptom scoring was conducted to evaluate the alleviating effect of the recombinant virus strain rPRV-Δ5-S1 on post-challenge diarrhea in piglets. A diarrhea scoring system (0 = normal; 1 = semi-formed feces; 2 = soft feces; 3 = watery with solid content; 4 = profuse watery diarrhea) was employed for quantitative analysis of clinical symptoms.

### 2.16 Histopathological analysis

For histopathological analysis, brain, lung, and spleen tissues were collected from mice euthanized at 14 dpc, while duodenum, jejunum, and ileum samples were collected from piglets euthanized at 14 dpc. Tissues were fixed in 4% paraformaldehyde, embedded in paraffin, sectioned, and stained with hematoxylin and eosin (H&E) for microscopic examination (Wuhan Servicebio Biotechnology Co., Ltd.).

### 2.17 Statistical analysis

Statistical analyses were performed using GraphPad Prism 8 software (GraphPad Software, Inc., USA). Data are presented as mean ± SD unless otherwise indicated. *In vitro* experiments represent at least three independent biological replicates.

For comparisons between two groups, an unpaired Student’s t-test was used. Comparisons among multiple groups at a single time point were performed using one-way analysis of variance (ANOVA) followed by Tukey’s multiple-comparison test. Longitudinal data, including body weight, antibody responses, rectal temperature, and viral shedding, were analyzed using two-way repeated-measures ANOVA followed by Tukey’s multiple-comparison test.

Clinical symptom scores and diarrhea scores were analyzed using nonparametric statistical methods. Comparisons between two groups were performed using the Mann–Whitney U test, while multiple-group comparisons were conducted using the Kruskal–Wallis test followed by Dunn’s multiple-comparison *post hoc* test where appropriate.

A *p* value < 0.05 was considered statistically significant. Statistical significance is indicated as follows: ∗*p* < 0.05, ∗∗*p* < 0.01, ∗∗∗*p* < 0.001, and ∗∗∗∗*p* < 0.0001.

Blinding was not implemented during animal allocation, vaccination, clinical monitoring, sample collection, or data analysis.

### 2.18 Anesthesia and euthanasia

All animal procedures involving anesthesia and euthanasia were performed in accordance with the AVMA Guidelines for the Euthanasia of Animals (2020 Edition).

Rabbits: Prior to euthanasia, rabbits were deeply anesthetized with an intraperitoneal injection of pentobarbital sodium (50 mg/kg body weight). Subsequently, a lethal dose of pentobarbital sodium (100 mg/kg) was administered intravenously via the marginal ear vein. Death was confirmed by the absence of heartbeat, respiratory arrest, and loss of corneal and pedal withdrawal reflexes.

Mice: According to the AVMA 2020 guidelines, euthanasia was performed by cervical dislocation carried out by trained personnel, and prior anesthesia was not required for this method. Death was confirmed by respiratory arrest and the absence of a heartbeat.

Piglets: Piglets were first sedated with an intramuscular injection of xylazine (2 mg/kg body weight). Then, a lethal dose of pentobarbital sodium (100 mg/kg) was administered intravenously via the auricular vein. Death was confirmed by the absence of heartbeat, respiration, and corneal reflex.

### 2.19 Ethics approval and consent to participate

The animal study was approved by the Ethics Committee at Northwest A&F University (approval number DY2022009). All operations involving animal experiments were handled according to the committee’s guidelines and were conducted in accordance with the local legislation, institutional requirements, and the Chinese National Standard ‘Laboratory Animal—Guideline for Ethical Review of Animal Welfare’ (GB/T 35892–2018). All euthanasia procedures were performed in accordance with the AVMA Guidelines for the Euthanasia of Animals (2020 Edition). The study was conducted in compliance with the ARRIVE guidelines for reporting animal research.

## 3 Results

### 3.1 Construction and characterization of recombinant viruses

The construction strategy is outlined in Figures 1A. First, using CRISPR/Cas9 mediated homologous recombination, we generated an intermediate recombinant, rPRV ΔgI US2 mCherry, from the parental PRV SX10 2015 strain by deleting the gI, gE, US2 and US9 genes and inserting the mCherry reporter at the combined deletion locus (Figures 1B). From this intermediate, we then constructed rPRV Δ5 by deleting the TK gene and inserting the eGFP cassette at the TK locus (Figures 1C). Separately, rPRV Δ5 S1 was generated by deleting the TK gene and inserting the PEDV S1 eGFP fusion gene at the same TK locus (Figures 1D). PCR analysis using specific primers for the deleted genes (TK, gI/gE, US9/US2) and the inserted markers (mCherry, eGFP, and PEDV S1) confirmed the correct genomic structures of both purified recombinants (Figures 1E).

To evaluate genetic stability, the recombinant viruses were serially passaged in PK-15 cells and analyzed at passages F5, F10, and F20. PCR analysis demonstrated persistent detection of the eGFP (or PEDV S1 in rPRV Δ5 S1) and mCherry genes, together with the continued absence of TK, gI/gE and US2/US9 genes throughout the evaluated passages (Supplementary Figure S1). These findings provide evidence of short-term genetic stability and maintenance of the expected recombinant genomic structure, but do not fully establish long-term genetic or expression stability.

Growth kinetics analysis showed that all three strains (PRV-SX10-2015, rPRV-Δ5, and rPRV-Δ5-S1) exhibited relatively low viral titers at 12 hpi (3.3–4.2 log10 TCID50/100 μL), followed by rapid replication. Peak viral titers were observed between 36 and 48 hpi, reaching 6.8–8.0 log10 TCID50/100 μL, and gradually declined to 6.5–7.2 log10 TCID50/100 μL at 72 hpi. Although recombinant strains exhibited slightly lower titers than the parental strain, replication kinetics were comparable (Figure 2A). Experiments were performed in three independent biological replicates. Western blot confirmed expression of the PEDV S1 protein (~92.3 kDa) in rPRV-Δ5-S1-infected cells (Figure 2B).

### 3.2 Enhanced safety of the quintuple-gene-deleted PRV vector in rabbits

**Figure 3:**
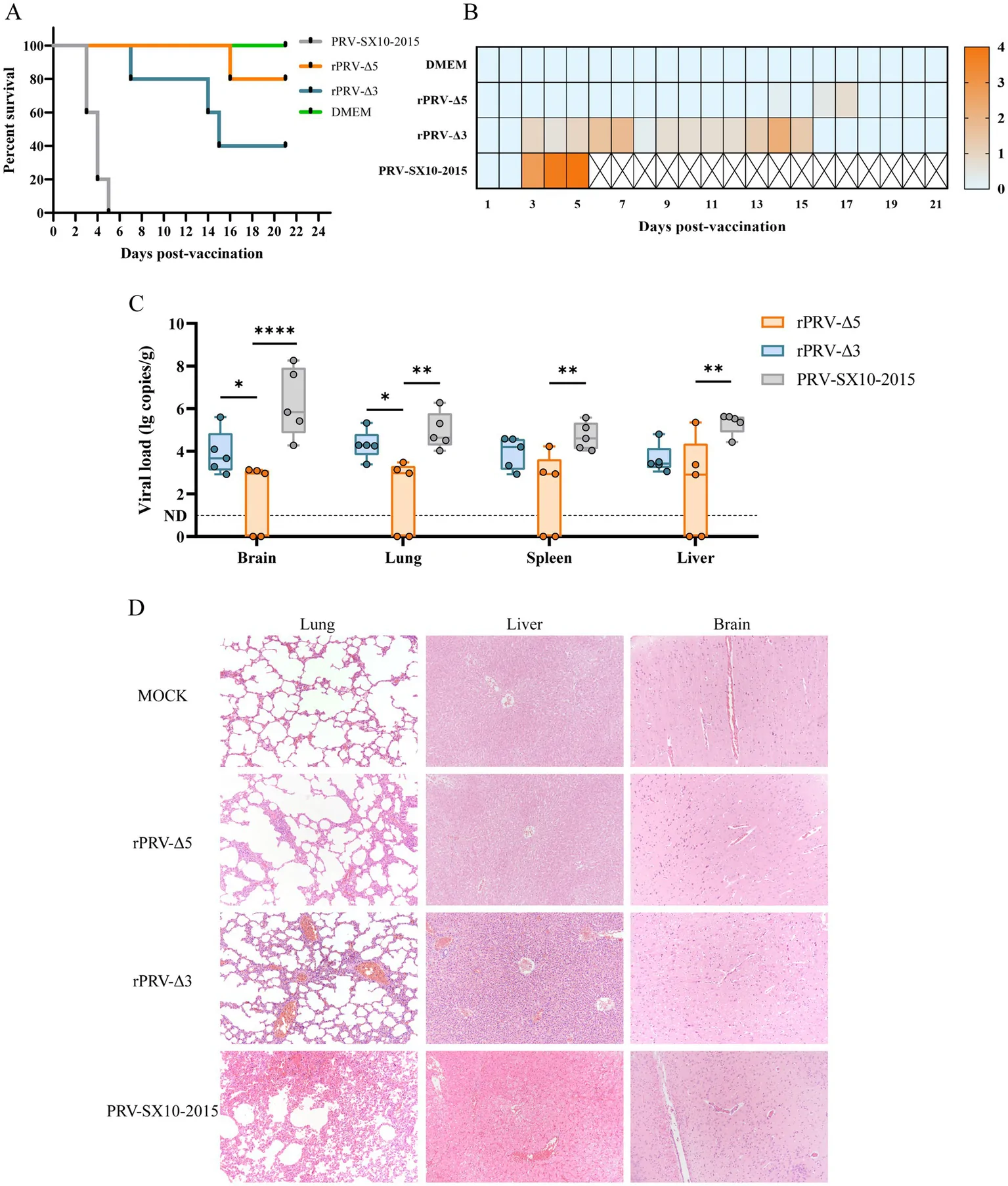
Safety evaluation of the quintuple-gene-deleted PRV vector in a rabbit model. *n* = 5 rabbits per group. **(A)** Survival outcomes of rabbits following immunization with different PRV vectors. **(B)** Heatmap of clinical neurological symptom scores in rabbits after immunization with different PRV vectors. **(C)** Viral loads in brain, lung, spleen, and liver tissues quantified by qPCR at necropsy. Data are presented as mean ± SD. Statistical significance was analyzed using one-way ANOVA followed by Tukey’s multiple-comparison test (∗*p* < 0.05, ∗∗ *p* < 0.01, ∗∗∗ *p* < 0.001, ∗∗∗∗ *p* < 0.0001; ns, not significant). **(D)** Histopathological analysis of liver, brain, and lung tissues collected at necropsy (H&E staining, magnification 200×).

To evaluate the safety, 30-day-old weaned rabbits were inoculated with PRV-SX10-2015, rPRV-Δ3, or rPRV-Δ5, respectively, and monitored for 21 days. All rabbits in the wild-type group died by 5 dpi (100% mortality), the rPRV-Δ3 group reached 60% mortality by 15 dpi, and the rPRV-Δ5 group showed only 20% mortality, with the first death on 16 dpi and no further deaths (Figure 3A).

Daily clinical symptom scoring (0 = no symptom; 4 = moribund/complete paralysis) revealed distinct pathogenicity profiles among the three groups (Figure 3B). In the rPRV-Δ5 group, infected rabbits showed minimal symptoms, with only one rabbit displaying moderate abnormality on 16–17 dpi. The rPRV-Δ3 group exhibited progressive symptom escalation, while the wild-type group showed rapidly severe symptom development. Heatmap patterns were consistent with survival observations.

The viral loads in brain, lung, spleen, and liver tissues were quantified (Figure 3C). PRV-SX10-2015-infected rabbits had high viral loads in all tissues, rPRV-Δ3-inoculated rabbits had intermediate loads, and the rPRV-Δ5 group showed the lowest viral loads, with several tissues testing negative. These results are consistent with the reduced pathogenicity.

Histopathological analysis at 21 dpi (Figure 3D) showed mild inflammatory infiltration in rPRV-Δ5-infected lungs and no obvious lesions in liver and brain. The rPRV-Δ3 group displayed moderate tissue damage, and the wild-type group exhibited extensive lung, liver, and brain lesions. These histopathological results are consistent with survival data, clinical observations, and viral load detection.

### 3.3 Immunogenicity and protective efficacy of the recombinant strains against PRV in mice

**Figure 4:**
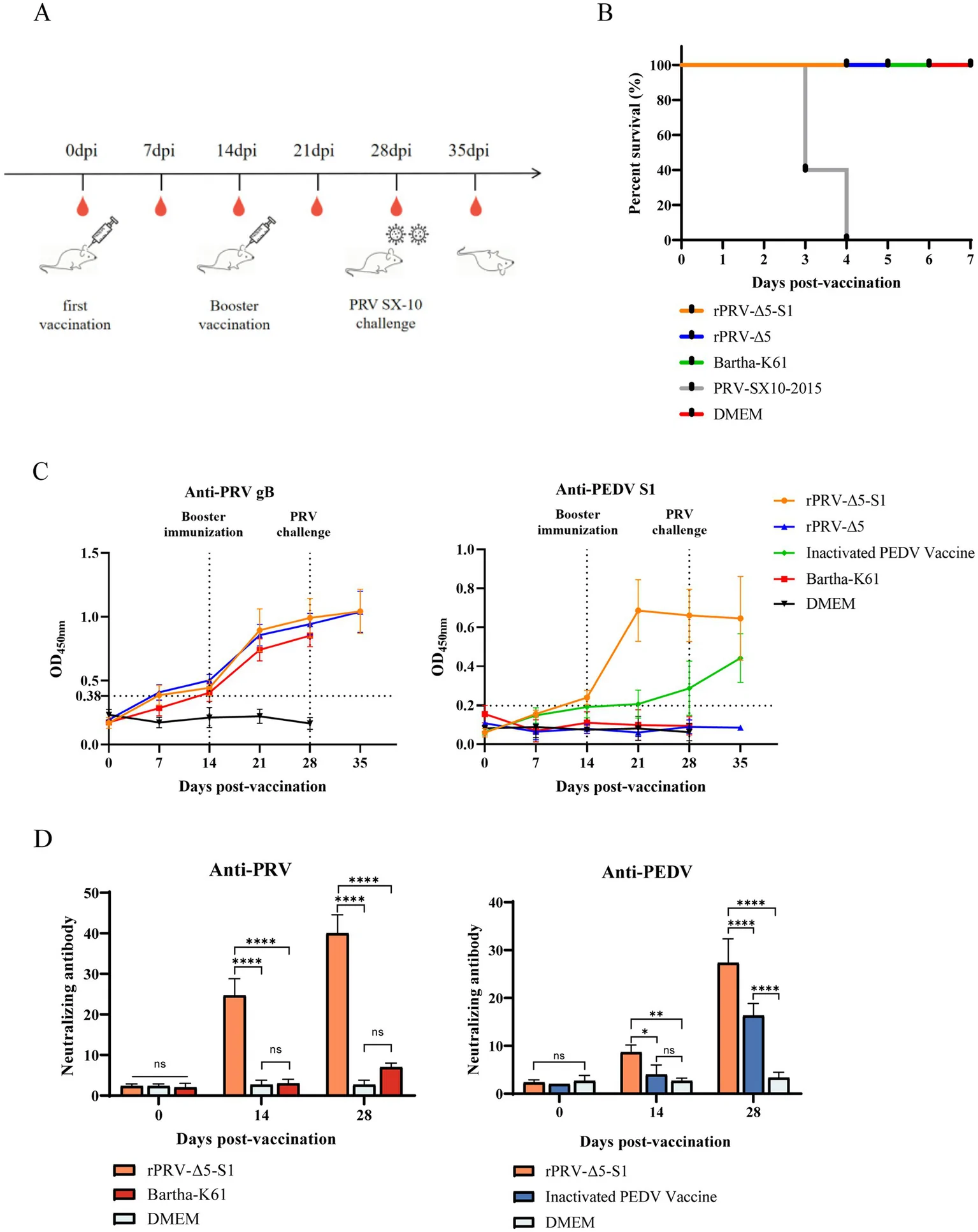
Evaluation of safety and immune protective efficacy in mice. *n* = 5 mice per group. **(A)** Schematic diagram of the immunization and challenge experiments in mice. **(B)** Survival status of mice during the 28-day post-vaccination safety observation period prior to PRV challenge. **(C)** Kinetics of PRV gB-specific and PEDV S1-specific IgG antibody responses in mice immunized with different PRV vectors. Data are presented as mean ± SD. Longitudinal antibody responses were analyzed using two-way repeated-measures ANOVA followed by Tukey’s multiple-comparison test. **(D)** Neutralizing antibody titers in mouse sera at 0, 14, and 28 days post-immunization against the PRV-SX-10-2015 strain and the PEDV-XN-2022 (G2b) strain. Statistical significance was analyzed using one-way ANOVA followed by Tukey’s multiple-comparison test (∗*p* < 0.05, ∗∗ *p* < 0.01, ∗∗∗ *p* < 0.001, ∗∗∗∗ *p* < 0.0001; ns, not significant).

Mice were immunized with rPRV-Δ5-S1, rPRV-Δ5, Bartha-K61, or DMEM. All mice in recombinant virus groups survived PRV challenge, while some control groups showed mortality (Figure 4B). No significant clinical abnormalities were observed.

PEDV S1- and PRV gB-specific antibodies were measured via ELISA (Figure 4C). The rPRV-Δ5-S1 group exhibited detectable PEDV S1-specific IgG, significantly higher than DMEM controls, Bartha-K61, and inactivated PEDV vaccine (*p* < 0.05–0.001). PRV gB antibody responses were comparable between recombinant groups and Bartha-K61 (*p* > 0.05) and higher than DMEM control group (*p* < 0.001).

**Figure 5:**
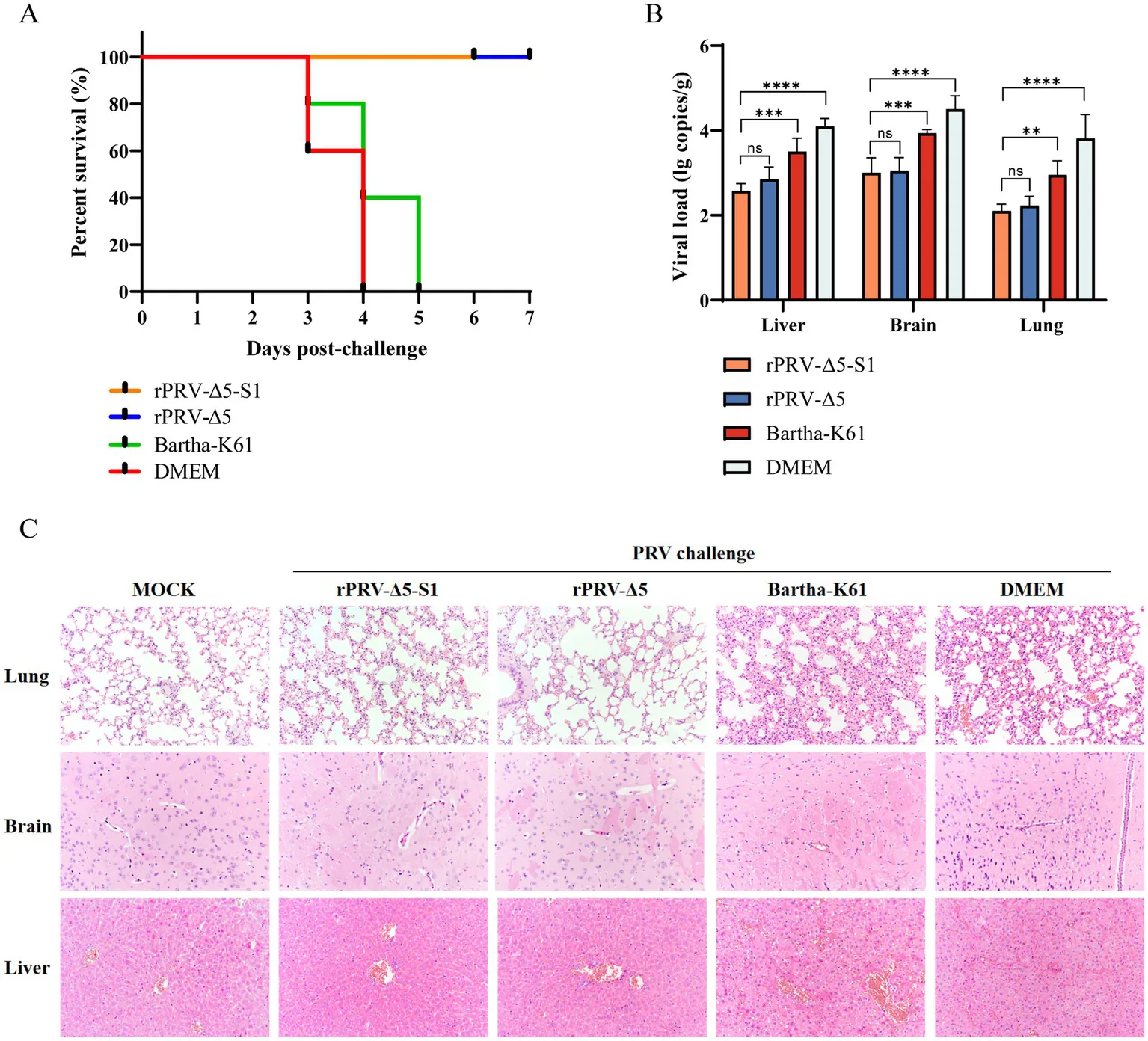
rPRV-Δ5 and rPRV-Δ5-S1 protects mice against lethal PRV infection. *n* = 5 mice per group. **(A)** Survival outcomes of mice challenged with PRV SX-10-2015, with DMEM serving as a control. **(B)** Viral loads in liver, brain, and lung tissues were quantified via qPCR following viral challenge. Data are presented as mean ± SD. Statistical significance was analyzed using one-way ANOVA followed by Tukey’s multiple-comparison test (∗*p* < 0.05, ∗∗*p* < 0.01, ∗∗∗*p* < 0.001, ∗∗∗∗*p* < 0.0001; ns, not significant). **(C)** Histopathological analysis of liver, brain, and lung from each group after challenge (H&E staining, 200×).

Neutralizing antibody titers (NT50) against PEDV and PRV were evaluated at 0, 14, and 28 dpi (Figure 4D). The rPRV-Δ5-S1 group showed detectable neutralizing antibodies against both viruses. Following booster immunization, PRV challenge was performed (Figure 5A). All mice vaccinated with recombinant viruses survived, and tissue viral loads were significantly reduced compared with control groups (Figure 5B). Histopathological analysis showed no significant organ damage in the recombinant vaccine groups (Figure 5C).

### 3.4 Preliminary evaluation of the protection efficacy of the rPRV-Δ5-S1 against PEDV in piglets

**Figure 6:**
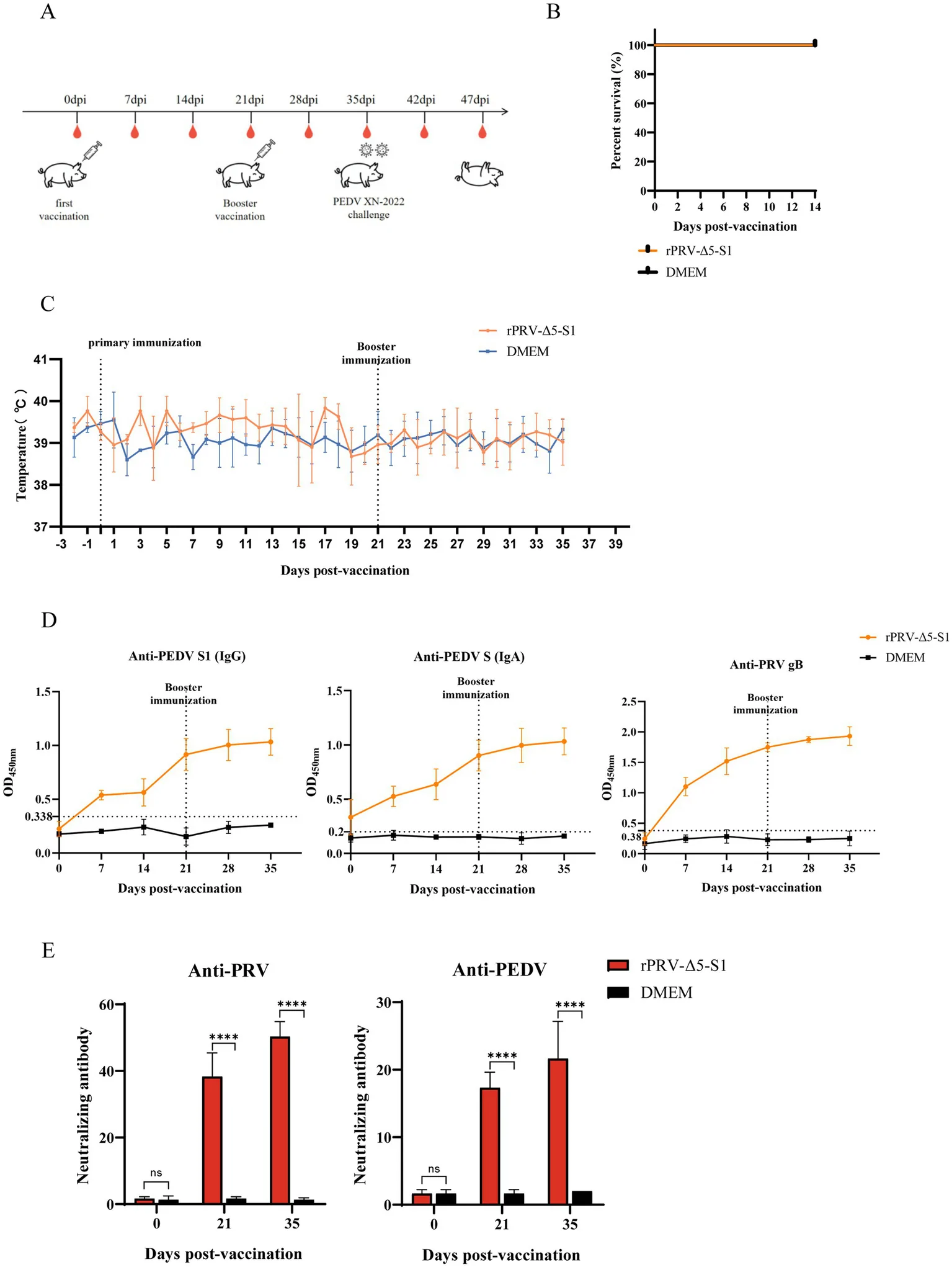
Evaluation of safety and immune protection effectiveness in piglets. *n* = 3 piglets per group. **(A)** Schematic diagram outlining the immunization and challenge experiments in piglets. **(B)** Survival status of vaccinated piglets throughout the study period. **(C)** Post-vaccination body temperature changes in piglets. Longitudinal temperature data were analyzed using two-way repeated-measures ANOVA followed by Tukey’s multiple-comparison test. **(D)** PEDV S1-specific IgG, PEDV S1-specific IgA, and PRV gB-specific antibody curves in immunized piglets. Data are presented as mean ± SD. Longitudinal antibody responses were analyzed using two-way repeated-measures ANOVA followed by Tukey’s multiple-comparison test. **(E)** Neutralizing antibody titers in piglets at 0, 21, and 35 dpi against PRV-SX-10-2015 and PEDV-XN-2022 (G2b). Statistical significance was analyzed using one-way ANOVA followed by Tukey’s multiple-comparison test. (∗*p* < 0.05, ∗∗ *p* < 0.01, ∗∗∗ *p* < 0.001, ∗∗∗∗*p* < 0.0001; ns, not significant).

Piglets were immunized with rPRV-Δ5-S1 and the safety, immune responses, and outcome after PEDV challenge were monitored. No mortality occurred, and the body temperatures were similar between vaccinated and DMEM groups (Figures 6A–C). qPCR detection of rectal swab samples revealed no detectable PRV.

PEDV S1-specific and PRV gB-specific antibodies were induced in vaccinated piglets, with IgG and IgA levels increasing post-primary and post-booster immunization (Figure 6D). NT50 assays showed detectable neutralizing antibodies against PRV and PEDV (Figure 6E).

**Figure 7:**
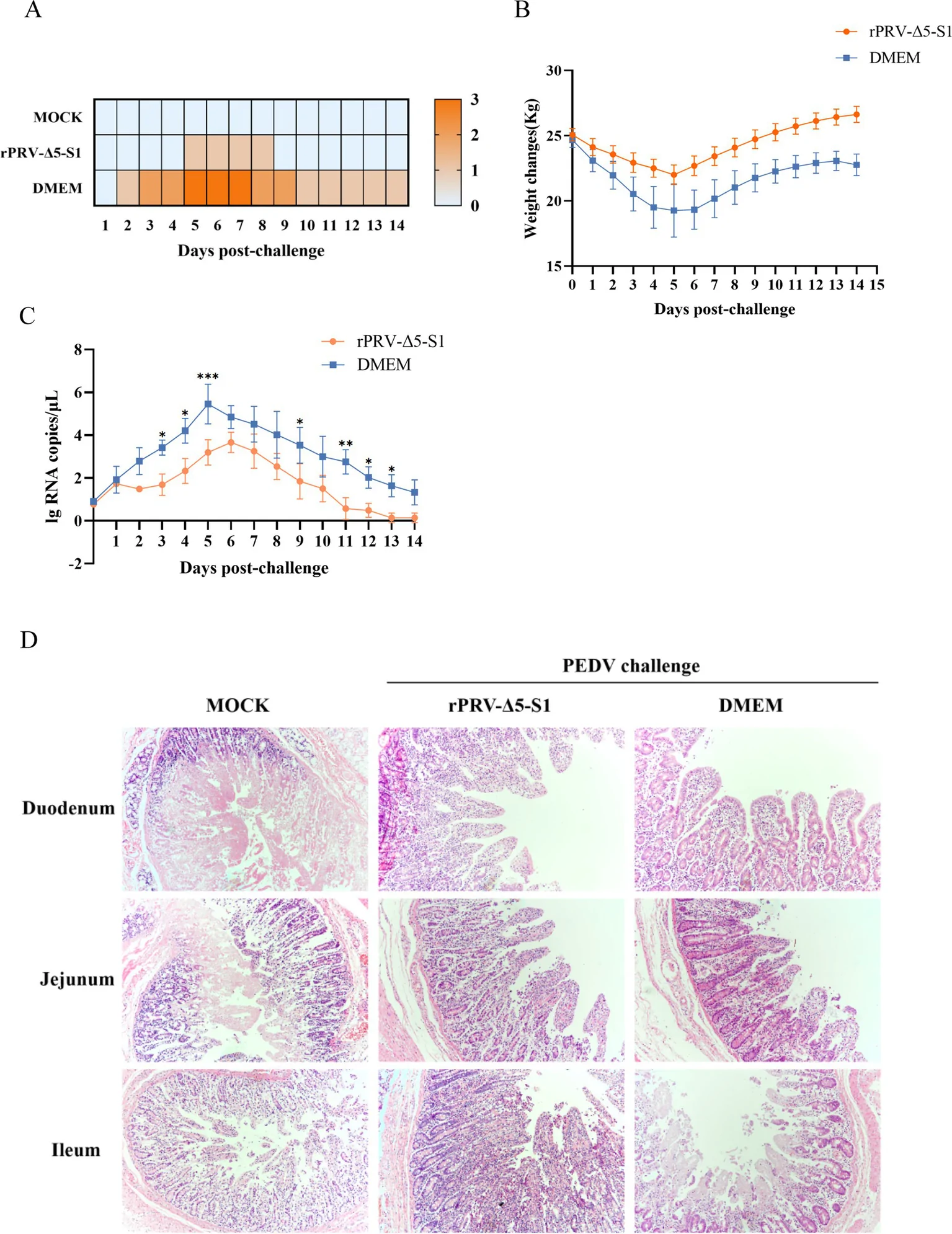
Protective efficacy of immunization with rPRV-Δ5-S1 against challenge with the variant strain PEDV-XN-2022 (G2b) in piglets. *n* = 3 piglets per group. **(A)** Heatmap of clinical diarrhea symptom scores in piglets post-challenge with PEDV-XN-2022 (G2b). **(B)** Post-challenge body weight changes in piglets. Data are presented as mean ± SD. Longitudinal body-weight data were analyzed using two-way repeated-measures ANOVA followed by Tukey’s multiple-comparison test. (∗*p* < 0.05, ∗∗ *p* < 0.01, ∗∗∗ *p* < 0.001, ∗∗∗∗ *p* < 0.0001; ns, not significant). **(C)** PEDV genome copy numbers in rectal swabs quantified by RT-qPCR following challenge. Data are presented as mean ± SD (*n* = 3 piglets per group). Statistical significance was analyzed using two-way repeated-measures ANOVA followed by Tukey’s multiple-comparison test (∗*p* < 0.05, ∗∗*p* < 0.01, ∗∗∗*p* < 0.001; ns, not significant). **(D)** Histopathological analysis of the duodenum, jejunum, and ileum in different groups post-challenge (H&E staining, 100×).

After PEDV challenge, compared with control group, vaccinated piglets exhibited reduced diarrhea severity and faster weight recovery (Figures 7A,B). Rectal swab viral loads peaked later and declined faster in vaccinated animals (Figure 7C). Histopathological analysis showed mild intestinal lesions in vaccinated piglets, whereas unvaccinated controls exhibited severe duodenal, jejunal, and ileal damage (Figure 7D). These results provide preliminary evidence that rPRV-Δ5-S1 may reduce disease severity and promote viral clearance following PEDV challenge.

## 4 Discussion

PEDV and PRV are two major pathogens that pose severe threats to the global swine industry. Previous studies have confirmed that PRV infection can compromise respiratory barrier integrity, increasing susceptibility to other pathogens, such as facilitating secondary infection with *Pasteurella multocida* (Zhang et al., 2024). PEDV infection causes acute, severe atrophic enteritis, leading to profuse diarrhea and dehydration (Lei et al., 2024; Su et al., 2024; Jung et al., 2020). Currently available commercial vaccines for PRV and PEDV are limited by insufficient immunogenicity, inadequate protection against emerging variants, or poor induction of effective mucosal immunity (Liang et al., 2020; Wei et al., 2024; Tan et al., 2021). To address this practical challenge, we utilized CRISPR/Cas9 technology to construct a recombinant viral vector based on a PRV variant with quintuple gene deletions (gI/gE/US2/US9/TK) and inserted the S1 gene from a prevalent PEDV G2b strain to generate a potential bivalent vaccine candidate, rPRV-Δ5-S1. This study systematically evaluated the safety of the recombinant vector in rabbits, PRV-related immunogenicity and protective efficacy in mice, and preliminary PEDV-related protection in piglets.

The present study evaluated the recombinant PRV vector in multiple complementary animal models. Rabbits were primarily used to assess vector safety, mice to evaluate PRV-related immunogenicity and protection, and piglets to obtain preliminary evidence of PEDV-related protection in the natural host. Collectively, these findings support further evaluation of rPRV-Δ5-S1 as a potential bivalent PRV/PEDV vaccine candidate.

Based on the PRV pathogenesis and immune evasion mechanisms, five genes (gI, gE, US2, US9, and TK) were selected for deletion. The gI/gE complex and US9 mediate axonal transport and viral spread in the nervous system; and their deletion blocks neuroinvasion (Tan et al., 2021; Scherer et al., 2020). US2 has been reported as a cGAS-STING antagonist (Kong et al., 2024; Wang et al., 2025; Lyu et al., 2018). Deletion of US2 may contribute to enhanced innate immune recognition; however, this was not directly tested in this study and should be considered a plausible interpretation rather than a demonstrated mechanism. TK is an essential gene for viral replication in non-dividing cells, and its deletion is well-established attenuation strategy for herpesvirus vaccine development (Tan et al., 2021; Wang et al., 2021). This quintuple-gene-deletion strategy draws on the natural deletion characteristics of the classic attenuated vaccine strain Bartha-K61 (which lacks gI, gE, US9, and US2) (Lomniczi et al., 1987), while additionally deleting TK to further improve safety. Furthermore, the vaccine vector was constructed on the backbone of a currently circulating PRV variant, ensuring antigenic match (Tan et al., 2021).

Rabbits are highly susceptible to PRV infection (Sehl and Teifke, 2020; Cao et al., 2022) and are used as a sensitive model for evaluating the residual virulence and safety of attenuated PRV vaccine candidates. Compared with the triple-gene-deleted strain rPRV-Δ3, the quintuple-gene-deleted vector rPRV-Δ5 showed higher survival (80% vs. 40%), milder clinical symptoms, lower viral loads, and minimal histopathological lesions. These findings indicated that the additional US2 and US9 deletions were associated with reduced virulence in rabbits. Based on this platform, insertion of the PEDV S1 gene generated rPRV-Δ5-S1, which was evaluated in mice and piglets. Our strategy is consistent with previous reports that showed quintuple-gene deletions improve safety compared to triple- or double-gene-deleted strains (Wang et al., 2025; Li et al., 2022).

In mice, rPRV-Δ5-S1 induced detectable PEDV S1-specific and PRV-neutralizing antibodies. Following PRV challenge, all mice immunized with rPRV-Δ5-S1 survived, with reduced viral loads and no histopathological damage, whereas Bartha-K61-immunized mice showed limited protection against the PRV-SX10-2015 challenge strain under the experimental conditions used in this study. These results suggest that the recombinant virus retained protective activity against PRV after S1 insertion.

In piglets, rPRV-Δ5-S1 demonstrated an acceptable safety profile, with no fever, mortality, or detectable PRV shedding post-immunization. The vaccine induced PEDV S1-specific IgG and IgA and PRV gB-specific IgG. Neutralizing antibody titers increased after booster immunization, indicating the induction of measurable humoral immune responses following booster immunization. Following PEDV challenge, vaccinated piglets exhibited reduced diarrhea severity, accelerated viral clearance, and improved weight recovery compared to controls. Histopathology showed only mild intestinal lesions in vaccinated animals, in contrast to severe lesions in unvaccinated controls. These findings provide preliminary evidence that rPRV-Δ5-S1 may reduce disease severity and promote viral clearance following PEDV challenge. Therefore, the piglet data should be interpreted as preliminary evidence of PEDV-related protection rather than definitive confirmation of field-level vaccine efficacy.

Several limitations of the present study should be acknowledged. First, the recombinant viruses retained eGFP and mCherry markers to facilitate identification during the experimental phase. Marker removal was not performed in this preclinical evaluation; however, for future commercial vaccine development, these markers could be excised using Cre/LoxP or similar systems. Second, the sample size of piglets was relatively small and only one age group was evaluated. Third, immunization studies in pregnant sows were not performed, and therefore maternal antibody transfer remains unknown. Fourth, only short-term immune responses were assessed. Finally, the PRV-PEDV co-infection model was not established in the present study.

## 5 Conclusion

In summary, the present study successfully constructed a recombinant quintuple-gene-deleted PRV vector expressing the PEDV S1 protein. Compared with the previously developed triple-gene-deleted PRV vector, the quintuple-gene-deleted backbone exhibited an improved safety profile in rabbits. The recombinant virus rPRV-Δ5-S1 retained immunogenicity, induced PRV- and PEDV-specific immune responses, and conferred protection against PRV challenge in mice. In piglets, vaccination provided preliminary evidence of PEDV-related protection following challenge. These findings support further evaluation of rPRV-Δ5-S1 as a potential bivalent PRV/PEDV vaccine candidate and provide a basis for future studies involving larger target-species trials and comprehensive efficacy assessment.

## Data Availability

The raw data supporting the conclusions of this article will be made available by the authors, without undue reservation.
