# Correction: Evaluation of a quintuple-gene-deleted PRV vector expressing PEDV S1: safety and immunogenicity in rabbits, mice, and piglets

**DOI:** 10.3389/fmicb.2026.1938063

**Published:** 2026-08-12

**Authors:** Frontiers Production Office

**Affiliations:** Frontiers Media SA, Lausanne, Switzerland

**Keywords:** CRISPR/Cas9 gene editing, immunogenicity, porcine epidemic diarrhea virus, pseudorabies virus, vaccine

Three figure cross-references were incorrectly provided in the published version of the article due to production errors. The figure references in Sections 2.10, 2.11, and 3.1 were incorrectly linked to the wrong figures.

A correction has been made to the Section 2.10, “*Immunization and challenge trial in mice*,” Section 2.11, “*Immunization and challenge trial in piglets*,” and Section 3.1, “*Construction and characterization of recombinant viruses*”:

**1. Section 2.10 Immunization and challenge trial in mice**

The figure reference in the sentence:

“A mouse model was employed to evaluate the safety and immunogenicity of rPRVs, with the immunization and challenge protection flow chart detailed in Figure 1A.” was incorrectly given as Figure 1A.

The correct figure reference is Figure 4A.

The corrected sentence reads:

“A mouse model was employed to evaluate the safety and immunogenicity of rPRVs, with the immunization and challenge protection flow chart detailed in Figure 4A.”

**2. Section 2.11 Immunization and challenge trial in piglets**

The figure reference in the sentence:

“The experimental design is illustrated in Figure 2A.” was incorrectly given as Figure 2A.

The correct figure reference is Figure 6A.

The corrected sentence reads:

“The experimental design is illustrated in Figure 6A.”

**3. Section 3.1 Construction and characterization of recombinant viruses**

The figure reference in the sentence:

“Although recombinant strains exhibited slightly lower titers than the parental strain, replication kinetics were comparable (Figure 4A).”

was incorrectly given as Figure 4A.

The correct figure reference is Figure 2A.

The corrected sentence reads:

“Although recombinant strains exhibited slightly lower titers than the parental strain, replication kinetics were comparable (Figure 2A).”

The original version of this article has been updated.

