# Supplementary material for: Evaluation of a quintuple-gene-deleted PRV vector expressing PEDV S1: safety and immunogenicity in rabbits, mice, and piglets
Source: Front Microbiol. 2026 Jul 8;17:1878909. doi: 10.3389/fmicb.2026.1878909 (PMC13388926; doi:10.3389/fmicb.2026.1878909)
Supplement: Supplementary file 1 [file Table_1.DOCX]

Supplementary Material

##
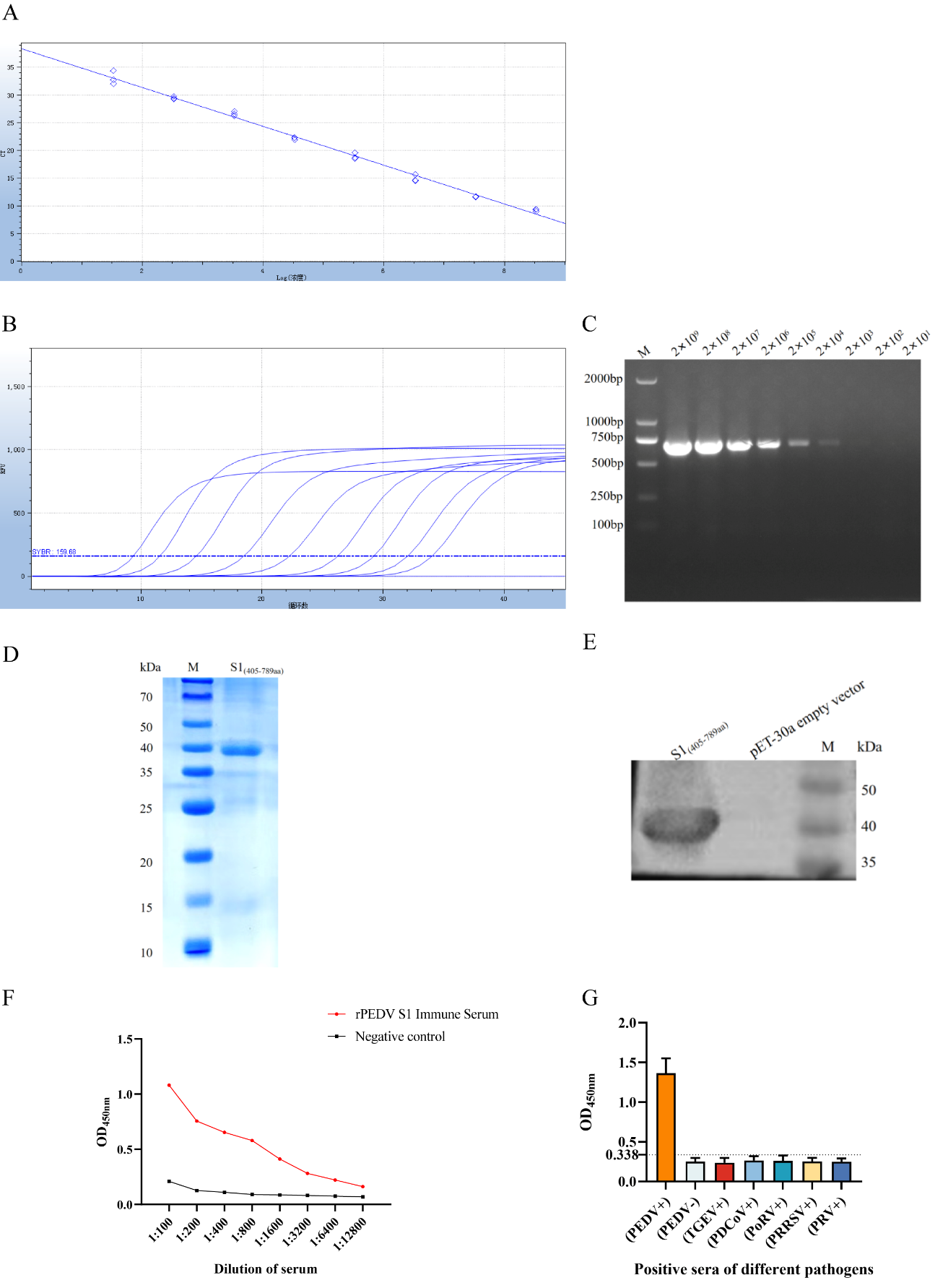
Supplementary Figures

**Supplementary Figure 1.** Development of the PEDV M gene quantitative RT-PCR assay, recombinant PEDV S1 protein expression and purification, preparation of PEDV S1-specific polyclonal antibodies, and establishment of the indirect ELISA are described in this Supplementary Material because these methods were developed as supporting analytical tools and were not the primary focus of the present study. **(A)** Standard curve of the SYBR Green I-based quantitative RT-PCR assay targeting the PEDV M gene. A recombinant pMD^TM^19-T plasmid containing the PEDV M gene fragment was used as the standard template, and 10-fold serial dilutions (10^1^-10^9^ copies/μL) were prepared for standard curve generation. **(B)** Amplification plots of the PEDV M gene quantitative RT-PCR assay using serially diluted standard plasmids. **(C)** Analytical sensitivity comparison between conventional RT-PCR and the PEDV M gene quantitative RT-PCR assay. The quantitative RT-PCR assay exhibited a detection limit of 3.34 × 10^2^ copies/μL, approximately 100-fold more sensitive than conventional RT-PCR. **(D)** SDS-PAGE analysis of recombinant PEDV S1 protein (amino acids 405–789) expressed in *Escherichia coli* BL21(DE3) and purified by Ni^2+^ affinity chromatography. The expected protein band was observed at approximately 41.4 kDa. **(E)** Western blot analysis of purified recombinant PEDV S1 protein using mouse-derived PEDV S1-specific polyclonal antibodies. Lysates from Escherichia coli carrying the empty pET-30a vector served as the negative control. **(F)** Determination of PEDV S1-specific polyclonal antibody titers by indirect ELISA. Serum samples were collected two weeks after the final immunization, and endpoint titers were determined using purified recombinant S1 protein as the coating antigen. **(G)** Specificity evaluation of the PEDV S1 indirect ELISA. PEDV-positive serum, PEDV-negative serum, and sera positive for transmissible gastroenteritis virus (TGEV), porcine delta coronavirus (PDCoV), porcine rotavirus (PoRV), porcine reproductive and respiratory syndrome virus (PRRSV), and pseudorabies virus (PRV) were tested to assess potential cross-reactivity. The assay specifically detected PEDV-positive serum without observable cross-reactivity with other swine viral pathogens.

The quantitative RT-PCR assay, recombinant PEDV S1 protein, polyclonal antibodies, and indirect ELISA described in this figure were developed as supporting tools for viral load determination and serological analyses performed in the present study.


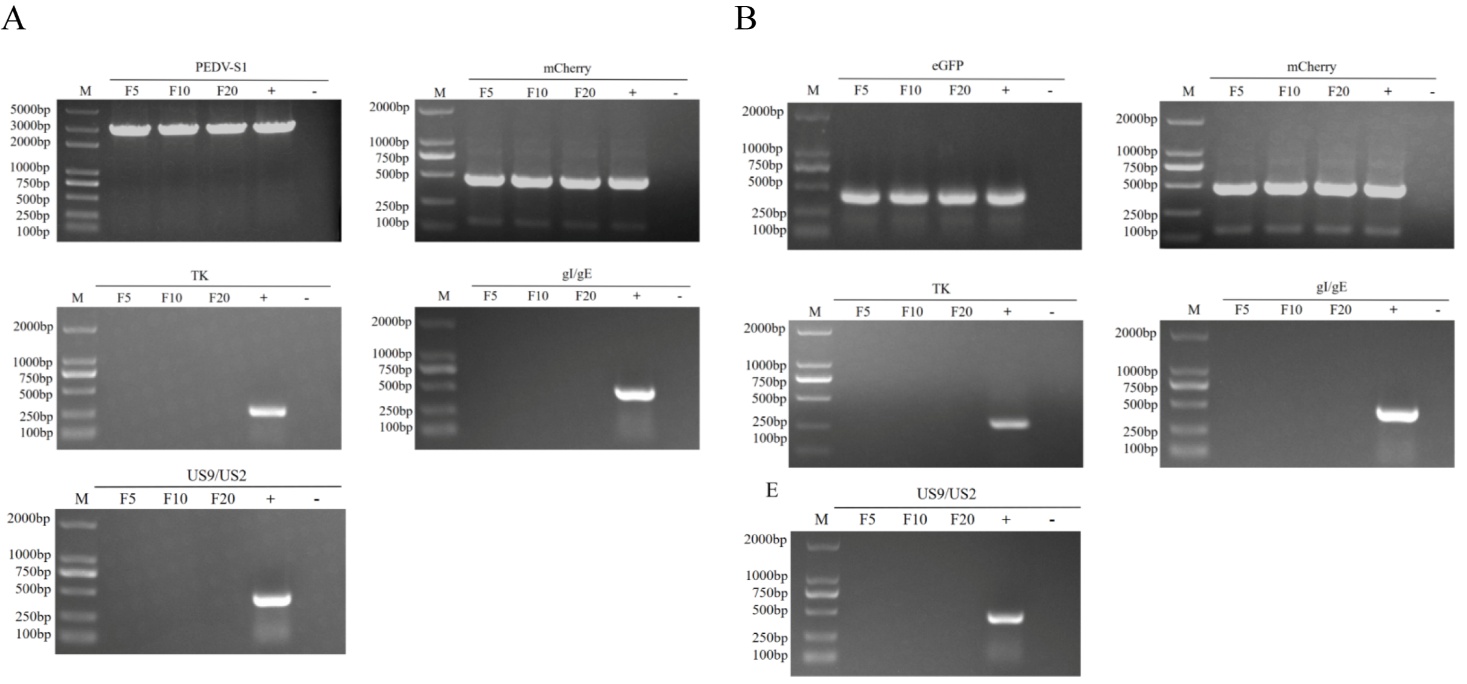
**Supplementary Figure 2.** Genetic stability testing of recombinant PRV. **(A)** PCR confirmation of stable foreign gene expression and virulence gene deletion in rPRV-Δ5-S1 after 20 passages. **(B)** PCR confirmation of stable foreign gene expression and virulence gene deletion in rPRV-Δ5 after 20 passages.
